# Harnessing technology and molecular analysis to understand the development of cardiovascular diseases in Asia: a prospective cohort study (SingHEART)

**DOI:** 10.1186/s12872-019-1248-3

**Published:** 2019-11-21

**Authors:** Jonathan Yap, Weng Khong Lim, Anders Sahlén, Calvin Woon-Loong Chin, Kenneth Michael Yun-Chi Chew, Sonia Davila, John Allen, Vera Goh, Swee Yaw Tan, Patrick Tan, Carolyn S. P. Lam, Stuart Alexander Cook, Khung Keong Yeo

**Affiliations:** 1 Cardiology, National Heart Centre Singapore, 5 Hospital Drive, Singapore, 169609 Singapore; 2 SingHealth Duke-NUS Institute of Precision Medicine, Singapore, Singapore; 3 Cancer and Stem Biology Program, Duke-NUS Medical School, Singapore, Singapore; 4 Cardiovascular and Metabolic Disorders Program, Duke-NUS Medical School, Singapore, Singapore; 5 Duke-NUS Medical School, Singapore, Singapore

**Keywords:** Primary prevention, Cardiovascular disease, Coronary artery disease, Epidemiology, Biomarkers, Ethnicity, Imaging

## Abstract

**Background:**

Cardiovascular disease (CVD) imposes much mortality and morbidity worldwide. The use of “deep learning”, advancements in genomics, metabolomics, proteomics and devices like wearables have the potential to unearth new insights in the field of cardiology. Currently, in Asia, there are no studies that combine the use of conventional clinical information with these advanced technologies. We aim to harness these new technologies to understand the development of cardiovascular disease in Asia.

**Methods:**

Singapore is a multi-ethnic country in Asia with well-represented diverse ethnicities including Chinese, Malays and Indians. The SingHEART study is the first technology driven multi-ethnic prospective population-based study of healthy Asians. Healthy male and female subjects aged 21–69 years old without any prior cardiovascular disease or diabetes mellitus will be recruited from the general population. All subjects are consented to undergo a detailed on-line questionnaire, basic blood investigations, resting and continuous electrocardiogram and blood pressure monitoring, activity and sleep tracking, calcium score, cardiac magnetic resonance imaging, whole genome sequencing and lipidomic analysis. Outcomes studied will include mortality and cause of mortality, myocardial infarction, stroke, malignancy, heart failure, and the development of co-morbidities.

**Discussion:**

An initial target of 2500 patients has been set. From October 2015 to May 2017, an initial 683 subjects have been recruited and have completed the initial work-up the SingHEART project is the first contemporary population-based study in Asia that will include whole genome sequencing and deep phenotyping: including advanced imaging and wearable data, to better understand the development of cardiovascular disease across different ethnic groups in Asia.

## Background

Cardiovascular disease (CVD) is a major cause of mortality and morbidity worldwide. Traditional large population-based studies in Western cohorts (e.g. Framingham Heart Study, Cardiovascular Health Study, Atherosclerosis Risk in Communities Study, Jackson Heart cohort) have formed the foundation of our initial knowledge on the risk factors of cardiovascular disease [1–4]. With advancements in technology, there have been many more exciting developments in our understanding of the development and prevention of CVD. Advances made in genomics, metabolomics, proteomics and data from wearables have the potential to deliver important new insights in the field of cardiology, especially after smaller datasets are combined into multi-domain, high-dimensionality “big data” and analyzed using novel machine learning techniques.

In the last couple of years, the American Heart Association (AHA) and Google Life Sciences (GLS) have forged a joint collaboration to use technology to tackle the scourge of CVD [5]. This collaboration will allow the scientific community to leverage on the technical capabilities and insights offered by Google Life Sciences. With the unique opportunity to access such resources, this project is forecasted to allow researchers to conceptualize and test new approaches [5]. Recent modern prospective population-based studies (Project Baseline, MURDOCK study) have also been developed to answer these questions with the aid of technology [6, 7]. Project Baseline aims to characterize participants across clinical, molecular, imaging, sensor, self-reported, behavioural, psychological, environmental and other health-related measurements from onsite visits, continuous data collection through sensor technology, and regular engagement via an online portal and mobile app [6]. The MURDOCK study aims to reclassify cardiovascular risk using integrated clinical and molecular biosignatures. However, these studies are based primarily in Western populations [7].

Ethnic differences in CVD exist [8–11]. The Multi-Ethnic Study of Atherosclerosis (MESA) study found distinct racial differences in the risk of incident heart failure [8]. The mechanisms are multifactorial with complex interactions between unmodifiable genetic factors and acquired risk factors. Often times, it has been difficult to distinguish the contributory role between socioeconomic and genetic factors towards development of disease with residual uncertainty regarding causality and pathogenic mechanisms [12, 13]. Currently, in Asia, there are no population-based studies that combine the use of conventional clinical information with advanced ‘discovery’-type molecular techniques, advanced quantitative cardiac imaging and wearable technologies to characterize the general community. We aim to harness these new technologies to understand the development of cardiovascular disease in Asia.

## Methods

### Study design and population

Singapore is a developed city state of 5.6 million people in Asia with well-represented diverse ethnicities including Chinese (74.3%), Malays (13.4%) and Indians (9.1%). The elderly ≥65 yr account for 12.4% of the population and the average life-expectancy is 82.9 years old [14].

The SingHEART study is the first multi-ethnic prospective population-based study of healthy Asians harnessing the latest technologies. In summary, healthy male and female subjects aged 21–69 years old without any prior cardiovascular disease (Ischemic heart disease, stroke, peripheral vascular disease) or diabetes mellitus will be recruited from the general population. The complete inclusion and exclusion criteria are found in the supplementary appendix (Additional file 1: Appendix 1). These patients are drawn from a cohort of healthy individuals who have already volunteered to the institutional Biobank program. Subjects fulfilling the inclusion criteria will be recruited from the public via advertisements (e.g. posters, local newspaper).

Written informed consent will be obtained from every subject and includes follow-up of up to 20 years, including agreement to permit tracking of outcomes via national and disease registries. Subjects will be informed of incidental abnormalities picked up during the tests. If genetic abnormalities are detected, there is an option for genetic counselling. All data will be anonymized for analysis.

Ethical approval was obtained from the Singhealth institutional review board.

### Objectives

This study aims to comprehensively characterize a healthy Asian population at baseline and at follow-up, using multiple modalities to aid in the understanding of the traditional and novel factors that contribute to the development of cardiovascular disease. Emphasis will be placed on understanding how these traditional and novel factors interact, as well as on elucidating the ethnic differences in the development of cardiovascular disease. The specific objectives are as follows:

- To characterize cardiovascular health in Asia by measuring multiple systems simultaneously and longitudinally.
- To assess lifestyle, diet, physical activity and sleep via traditional questionnaire surveys and wearable technologies and their impact on the development of cardiovascular disease.
- To characterize baseline genetic, metabolic and advanced cardiac imaging profiles in healthy individuals and the identification of novel markers influencing the development of cardiovascular disease with the potential for therapeutics.
- Validate patient-reported sleep/physical activity and that derived by wearables, study the impact of physical activity on cardiac structure, investigate relationship between calcium score and lifestyle factors, etc.
- To study the development and progression of cardiovascular disease in these patients to help develop new preventive, diagnostic and predictive tools for the Asian population
- To understand the differential effects of ethnicity on the development of cardiovascular disease
- To use both traditional statistical analysis and newer methodologies (e.g. machine learning) to process and analyze the data

### Study protocol

Each subject will undergo the following investigations at baseline and at specific intervals on follow-up for up to 20 years, as described in Table 1.

**Table 1:** List and timeline of investigations for SingHEART

Investigations	Baseline	10y	20y
Questionnaire	X	X	X
Basic blood investigations ^a^	X		
Electrocardiogram	X	X	X
Ambulatory BP monitor	X	X	
Continuous ECG monitoring	X	X	
Activity and sleep tracker	X	X	
Calcium Score	X		
Cardiac MRI	X		
Genomics and lipidomics	X		

^a^ Includes full blood count, renal and liver function, fasting lipids and glucose

#### Questionnaire

The questionnaire will include sections on demographics, socioeconomic status, medical history, lifestyle, diet and exercise, quality of life as assessed by the EQ-5D-3 L, Pittsburgh sleep quality index and International Index of Erectile Function (IIEF)- 5 (only for males). See supplementary appendix for the complete questionnaire (Additional file 2: Appendix 2).

#### Basic blood investigations

This will include full blood count, renal and liver function, fasting lipids and fasting glucose. As diabetes mellitus is an exclusion criterion, HbA1c will not be measured routinely. Samples are biobanked for future use through the NHCS biobank.

#### Electrocardiogram

A standard 12-lead resting ECG will be recorded. Variables studied will include rate, rhythm, axis, conduction intervals, morphologies (including QRS, S-T and T wave abnormalities) and arrhythmias.

#### Ambulatory BP monitor

Ambulatory blood pressure monitoring will be performed for 24 h via a cuff-monitoring (Spacelab Healthcare Model 90,227/90217A). Data collected will include the systolic, diastolic and mean blood pressure during the day and night, as well as data on dipping.

#### Continuous ECG monitoring

Continuous ECG monitoring will be performed for 24 h–72 h via a wearable, multi-lead ECG monitoring patch which stores data for up to 3 days (ePatch® ECG recorder AMS3000). Data collected will include variations in heart rate and the various types of arrhythmias present.

#### Activity and sleep tracker

A commercially available wearable fitness device able to track heart rate, physical activity, exercise and sleep will be used. The participants will wear this activity tracker for 5–7 days. Currently, the study uses the Fitbit Charge HR. Data for each subject will be downloaded from the Fitbit Application Programming Interface (https://dev.fitbit.com/reference/web-api/quickstart)_._ Step counts are available at two levels; intraday step counts in 15-min intervals and daily totals. Intraday HR data are available at 5-min intervals, along with confidence levels. Intraday sleep tracking data containing details of each sleep session will also be recorded.

#### Calcium score

Electron beam CT scanning using contiguous 3-mm slices during a single breath hold will be performed by a 320-slice CT scanner (Toshiba Aquilion ONE) with the following parameters: tube voltage 120kVp, tube current 200-400 mA (dependent on patient size and shape as visually assessed by the radiographers), gantry rotation time 350 ms and 3 mm section thickness. The non-contrast scan will be volume prospective and ECG-gated and synchronized to the RR interval with a scan time of 100 ms/slice. A calcified lesion will be defined as at least two contiguous voxels with an attenuation coefficient > 130 Hounsfield units. Coronary calcium scores will be calculated as previously described [15] via Vitrea Workstation.

#### Cardiac MRI

Cardiovascular phenotyping using cardiovascular magnetic resonance (CMR) will be performed in all healthy volunteers (3 T Ingenia, Philips or 1.5 T MAGNETOM Aera, Siemens). Conventional balanced steady-state free precession cine images of the vertical and horizontal long-axis planes and the sagittal LV outflow tract view will be acquired. Short-axis cines will be obtained from the mitral valve annulus to the apex (8 mm slices with 2 mm gap). In addition, a single breath hold 3D LV short axis stack will also be acquired in the same orientation. Aortic flow will be assessed using velocity-coded phase contrast imaging. Cardiac volumes, left ventricular mass, atrial sizes and aortic root will be measured in all patients using the CMR 42 software (Circle Cardiovascular Imaging). Normal CMR reference ranges in the Asian population were recently published using standardized protocols in our Image Analysis Laboratory [16].

#### Lipidomics studies

20 μl of lipid internal standard mix and 10 μl of 14:0 phosphatidylcholine will be added to 100 μl of serum in a microcentrifuge tube. After an equilibration period of 30 s, 1.2 ml of HPLC-grade methanol will be added to the mixture, followed by vortexing. The mixture will be incubated at 50 °C for 10 min, followed by centrifugation to pellet the precipitated protein. The supernatant will then be removed and placed in a clean microcentrifuge tube for drying under nitrogen gas. 100 μl of methanol will be used to reconstitute the dried extract. The reconstituted lipid solution will be separated using a LC-MS (liquid chromatography – mass spectrometry) system (Agilent 1260) and a Thermo Scientific Accucore HILIC column (100 × 2.1 mm; particle size 2.6 μm). Mobile phase A consists of acetonitrile/water (95:5) with 10 mM ammonium acetate, pH 8.0 and mobile phase B consists of acetonitrile/water (50:50) with 10 mM ammonium acetate, pH 8.0. For the separation, the column will be equilibrated with 100% mobile phase A, increasing to 20% mobile phase B in 5 min, then held for 5 min. The column will finally be re-equilibrated with 100% mobile phase A for 5 min. Finally, mass spectrometry and data acquisition will be performed using an Agilent 6430 triple-quadrupole mass spectrometer [17].

#### Genomics studies

Whole genome sequencing will be done in Illumina HiSeq X sequencers at 30X coverage. 1.0 micrograms of DNA per sample will be used for library preparation. Sequencing libraries will be generated using the Truseq Nano DNA HT sample preparation kit (Illumina, SUA) and idenx codes will be added to each sample. Briefly, the genome will be fragmented by sonication to a size of 350 bp, and the DNA fragments will be end-polished, A-tailed and ligated with full-length adapter for Illumina sequencing with further PCR amplification. Lastly, PCR products will be purified (AMPure XP system) and the libraries analysed for size distribution by Agilent 2100 Bioanalyzer and quantified using real-time PCR.

### Outcomes

Outcomes will be tracked 6 monthly for 20 years via review of medical records. The outcomes studied will include mortality and cause of death, myocardial infarction, stroke, malignancy, heart failure, and the development of comorbidities (e.g. ischemic heart disease, peripheral vascular disease, diabetes mellitus, renal failure, etc.). Patients provided explicit consent for the matching of outcomes against databases and registries. Information on mortality, myocardial infarction, stroke, chronic kidney disease and malignancy will be obtained from national state-mandated registries (e.g. Singapore Myocardial Infarction Registry, Singapore Stroke Registry, Singapore Renal Registry, Singapore Cancer Registry, Singapore Renal Registry, etc).

### Study management

The SingHEART Steering Committee is responsible for the overall conduct of the study. The protocol design and execution of the study is entirely under the oversight of the SingHEART Steering Committee, and the funding sources have no access to patient-specific data. Because volunteers are drawn from the Biobank cohort, the SingHEART program adheres to protocols managing patient data anonymization, patient confidentiality/privacy, incidental finding management; and biosample/genomic DNA samples. Data is entered electronically into a pre-designed database by trained study coordinators and the accuracy of the data will be regularly audited. Requests to use the data or biospecimens for research will require approval from the steering committee in accordance with standardized procedures. The study team meets once every month to review study progress and to address any concerns raised by the study coordinators.

### Statistics

The initial target enrolment is 2500 based on feasibility and initial funding availability, with the opportunity to extend recruitment with further funding. Multiple linear regression and logistic regression analyses using generalised linear models will be performed to study the relationships between factors. Odds ratios, 95% confidence intervals and *p*-values will be reported. Cox-proportional hazards models will be used to analyse longer-term health outcomes and hazards ratios reported. Multivariate adjustment for confounders will be performed. Machine learning methodology appropriate for high-dimensionality datasets (e.g. deep learning systems and neural networks, classification techniques involving decision trees and probabilistic prediction) will also be performed in tandem with conventional statistical analysis.

## Preliminary results

From October 2015 to May 2017, an initial 400 subjects have been recruited and all have completed the baseline investigations as documented above. Table 2 describes the baseline characteristics of the initial subjects. Further recruitment is currently ongoing and as of February 2018, 683 patients have been recruited.

**Table 2:** Selected baseline characteristics of initial cohort (*n* = 400)

Demographics	
Age, years	46 ± 13
Male, *n* (%)	175 (44)
Ethnicity, n (%)	
Chinese	358 (90)
Indian	11 (3)
Malay	19 (5)
Others	12 (3)
BMI (kg/m^2^)	24 ± 4
BSA (m^2^)	1.7 ± 0.2
Waist circumference (m)	83 ± 11
Hip circumference (m)	96 ± 10
Alcohol history, n (%)	152 (38)
Smoking history, n (%)	32 (8)
Income, n (%)	
< $3000	155 (40)
$3000–4999	109 (28)
> $5000	121 (31)
Occupation, n (%)	
Involving manual labour	28 (7)
Not involving manual labour	290 (74)
Not working	74 (19)
At least university education, *n* (%)	206 (52)
Married, *n* (%)	266 (67)
Questionnaire	
Exercise per week (min) (median and inter-quartile range)	45 (30,60)
Servings of vegetable a day	1.9 ± 0.9
Servings of fruits a day	1.4 ± 0.9
Cups of coffee a day	1.5 ± 0.9
Blood investigations	
Hemoglobin (g/dL)	13.6 ± 1.5
Creatinine (μmol/L)	69 ± 16
Fasting glucose (mmol/L)	5.3 ± 0.5
High-density Lipoprotein (mmol/L)	1.49 ± 0.34
Low-density Lipoprotein (mmol/L)	3.37 ± 0.8)
Electrocardiogram	
PR (ms)	160 ± 22
QRS (ms)	89 ± 11
QTc (ms)	424 ± 22
Vitals monitoring	
24 h Systolic blood pressure (mmHg)	116 ± 13
Systolic blood pressure (mmHg)	128 ± 18
Systolic blood pressure dipping (mmHg)	9 ± 6
24 h Diastolic blood pressure (mmHg)	73 ± 9
Diastolic blood pressure (mmHg)	78 ± 13
Diastolic blood pressure dipping (mmHg)	11 ± 7
Mean arterial pressure (mmHg)	88 ± 9
Mean arterial pressure dipping (mmHg)	9 ± 7
Mean heart rate (bpm)	72 ± 8
Mean pulse pressure (mmHg)	43 ± 7
Calcium score (Agatston units)	39 (238)
Cardiac MRI	
Left ventricular ejection fraction (%)	63 ± 6
Left ventricular end diastolic volume index (ml/m^2^)	71 ± 12
Left ventricular end systolic volume index (ml/m^2^)	27 ± 7
Left ventricular mass index (g/m^2^)	43 ± 13

N.B. Continuous variables presented as mean ± SD and categorical variables as n (%) unless otherwise indicated

## Discussion

In recent times, there has been a revolution in healthcare brought about by quantum leaps in technology. This has brought with it the unprecedented opportunity to better characterise and manage diseases and improve healthcare. Different advancements include the use of “artificial intelligence” and “deep learning”, the development of molecular medicine with improvements in genomic, metabolomic and proteomic screening and the use of devices like wearables which can constantly monitor various physiological data.

As mentioned earlier, there have been several collaborations primarily in Western cohorts to tap on this technology boom to better characterise cardiovascular diseases [5–7]. The AHA and GLS aim to leverage on the technology and analysis by Google technical capabilities and insights offered by Google Life Sciences to analyze the various aspects of cardiovascular diseases [5]. Project Baseline, a collaboration between Duke, Stanford and Verily, aims to collect broad phenotypic data across multiple domains including continuous sensors from thousands of participants, harnessing the power of technology to do so [6]. The SingHEART study aims to be one of the most comprehensive phenotypic studies of cardiovascular diseases in the world. We will characterize phenotypes on multiple fronts including a) questionnaires on lifestyle, diet, exercise data, etc. b) detailed cardiac imaging and investigations including cardiac MRI, 24 h ECG and blood pressure monitoring, and calcium score c) wearables for constant physiological monitoring and d) genomic and lipidomic screening. One of the unique features of SingHEART is the *pre-hoc* intention to pool data across multiple domains into larger datasets suitable for analysis by machine learning algorithms. Early work from this project on the relationship between wearable data and cardiovascular risk factors in normal volunteers have recently been published based on this approach [17] which will provide the foundation for further insights and understanding into the development and prevention of CVD in this cohort.

Beyond the use of technology to characterize cardiovascular diseases, SingHEART affords the opportunity to better elucidate differences across different Asian ethnicities. Ethnic differences have been known to exist across the spectrum of cardiovascular diseases. With regards to heart failure (HF), the Multi-Ethnic Study of Atherosclerosis (MESA) study found the risk of incident HF to be different amongst races [8]. Prior studies in Asian patients with HF showed increased adverse outcomes in Malays and Indians compared to Chinese [9]. For coronary heart disease (CHD), Whites appear to have higher risk of CHD compared to blacks, Latinos and Asians [8]. A local study found Indians to have the greatest incidence of myocardial infarction but Malays to have the highest mortality rate [11]. SingHEART aims to understand the mechanistic causes behind these ethnic differences. Furthermore, the overlapping influence of genetics and environmental or behavioral factors on the development of disease may be difficult to tease out in the traditional setting [12, 13]. With growth of technology and genetic research, this divide has been narrowed [12]. Studies like SingHEART with detailed genotypic, phenotypic and socioeconomic characterization will allow the opportunity to study this differentiation in greater depth.

The SingHEART project is the first contemporary population-based study in Asia that will allow us to harness up-to-date technology to better understand the development of cardiovascular disease across different ethnic groups. This will provide invaluable opportunities to develop pertinent public health prevention strategies, guide allocation of healthcare resources, improve the diagnosis and management of cardiovascular disease, and serve as a foundation for future research.

## Supplementary information

**Additional file 1.** Appendix 1. Complete inclusion and exclusion criteria for SingHEART study.

**Additional file 2.** Appendix 2. SingHEART Questionnaire.

## Data Availability

The datasets generated and/or analyzed during the current study are not publicly available due the ongoing nature of this study, but are available from the corresponding author on reasonable request.

## Abbreviations

AHA: American Heart Association
CHD: Coronary heart disease
CMR: Cardiovascular magnetic resonance
CT: Computed tomography
CVD: Cardiovascular disease
ECG: Electrocardiogram
GLS: Google life sciences
HF: Heart failure
MESA: Multi-ethnic study of atherosclerosis
MRI: Magnetic resonance imaging
NHCS: National Heart Centre (Singapore)
PRISM: Institute of Precision Medicine
