# Supplementary material for: Harnessing technology and molecular analysis to understand the development of cardiovascular diseases in Asia: a prospective cohort study (SingHEART)
Source: BMC Cardiovasc Disord. 2019 Nov 21;19:259. doi: 10.1186/s12872-019-1248-3 (PMC6873552; doi:10.1186/s12872-019-1248-3)
Supplement: Supplementary file 1 — Additional file 1. Appendix 1. Complete inclusion and exclusion criteria for SingHEART study. [file 12872_2019_1248_MOESM1_ESM.pdf]

National Heart Centre Singapore
SingHealth

**Tel: (65) 6704 8000**
**Fax: (65) 6844 9030**
National Heart Centre Singapore
5 Hospital Drive
Singapore 169609
www.nhcs.com.sg
Reg No 199801148 C

## Inclusion & Exclusion Criteria Checklist

**Study Title: SingHEART – Effects of physical activity, ambulatory blood pressure and calcium score on cardiovascular health in normal people (2015/2601)**

***Sub-study of: Molecular and Imaging Studies of Cardiovascular Health and Disease (IRB 2013/605/C)***

Date of screening : ______________________________

Subject initial : ______________________________

Subject ID : ______________________________

Affix Subject Label

| No. | Inclusion Criteria | Yes | No |
|---|---|---|---|
| 1. | Aged 21-69 years old | | |
| **No.** | **Exclusion Criteria** | **Yes** | **No** |
| 1. | Previous myocardial infarction (MI), inclusive of ST-elevation MI (STEMI) and non-ST-elevation MI (NSTEMI) | | |
| 2. | Known coronary artery disease – prior coronary revascularization | | |
| 3. | Known documented peripheral arterial disease | | |
| 4. | Previous stroke<br>a. Stroke is defined as new focal neurological deficit persisting more than 24hours | | |
| 5. | More than ongoing use of ≥2 anti-hypertensive agents | | |
| 6. | Prior history of cancer (excludes pre-cancerous lesions) | | |
| 7. | Expected life expectancy less than 1 year | | |
| 8. | Known definite diabetes mellitus or on treatment for diabetes mellitus | | |
| 9. | Known autoimmune disease or genetic disease | | |
| 10. | Known endocrine disease on treatment | | |
| 11. | Psychiatric illness | | |
| 12. | Asthma or chronic lung disease requiring long term medications or oxygen | | |
| 13. | Chronic infective disease, including tuberculosis, hepatitis B and C; and HIV | | |
| 14. | Inability to comply with study protocol | | |
| 15. | Any other acute or chronic medical or physical condition deemed by the investigator to affect study outcomes | | |

***Subject is unable to be recruited into study if 'yes' was checked for any of exclusion criteria.***

☐ *Tick if screen failure*

*Other reason(s) for screen failure* : ______________________________________________

Name of Person Obtaining Consent : ____________________________________

Signature of Person Obtaining Consent : ____________________________________

Date : ____________________________________
