# Supplementary material for: Harnessing technology and molecular analysis to understand the development of cardiovascular diseases in Asia: a prospective cohort study (SingHEART)
Source: BMC Cardiovasc Disord. 2019 Nov 21;19:259. doi: 10.1186/s12872-019-1248-3 (PMC6873552; doi:10.1186/s12872-019-1248-3)
Supplement: Supplementary file 2 — Additional file 2. Appendix 2. SingHEART Questionnaire. [file 12872_2019_1248_MOESM2_ESM.pdf]

SINGHEART Patient Questionnaire

DEMOGRAPHICS

1) Race?

☐Chinese ☐Malay ☐Indian ☐Others: ______

2) Country of birth?

☐Singapore ☐Others: ______

3) Marital status?

☐Married ☐Single ☐Widowed ☐Separated/divorced

4) Religion?

☐Free thinker ☐Christian/Catholic ☐Buddhist ☐Taoist ☐Islam ☐Hindu

☐Judaism ☐Others:_____

5) Highest education level?

☐ No formal education ☐Primary ☐Secondary/High school ☐Polytechnic

☐ITE ☐A-Level ☐Degree/University or higher

6) Type of housing currently living in

☐1 or 2 Room HDB:	☐Owned or ☐ Rented?
☐3 Room HDB:	☐Owned or ☐ Rented?
☐4 Room HDB:	☐Owned or ☐ Rented?
☐5 Room HDB	☐Owned or ☐ Rented?
☐HUDC:	☐Owned or ☐ Rented?
☐Condominium:	☐Owned or ☐ Rented?
☐Landed property:	☐Owned or ☐ Rented?
☐Others: __________ :	☐Owned or ☐ Rented?

7) Occupation?

☐Unemployed/retired ☐Blue collared (involving manual labour)

☐White collared (office work, professionals) ☐Pink collared (service industry)

8) Monthly income?

☐<$1000/Not working ☐$1000-1999 ☐$2000-2999 ☐$3000-4999

☐$5000-6999 ☐$7000-8999 ☐$9000 and more

9) How many siblings?

___ brother(s) ___ sister(s)

10 How many children?

___ son(s) ___ daughter(s)

MEDICAL HISTORY

11) Do you have a family history (siblings OR father/mother OR children) of: (tick all that apply)

☐Diabetes	Who? _____	What age?_____
☐Hypertension (high blood pressure)	Who? _____	What age?_____
☐Hyperlipidemia (high cholesterol)	Who? _____	What age?_____
☐Heart attack/heart blockage	Who? _____	What age?_____
☐Heart failure	Who? _____	What age?_____
☐Other heart disease: (specify:___________)	Who? _____	What age?_____
☐Sudden unexpected death	Who? _____	What age?_____

12) Do you have any of the following medical conditions (tick all that apply)

☐Coronary artery disease	Duration _______ (years)
☐Heart attack	Duration _______ (years)
☐Previous stenting	Duration _______ (years)
☐Previous coronary artery bypass	Duration _______ (years)
☐Heart failure	Duration _______ (years)
☐Previous stroke	Duration _______ (years)
☐Diabetes mellitus	Duration _______ (years)
☐Hypertension (high blood pressure)	Duration _______ (years)
☐Hyperlipidemia (high cholesterol)	Duration _______ (years)
☐Cancer, please specify: __________	Duration _______ (years)

☐Asthma Duration _______ (years)

☐Kidney failure on dialysis Duration _______ (years)

☐Chronic lung disease Duration _______ (years)

☐Hepatitis B/C Duration _______ (years)

☐Others, please specify: _________ Duration _______ (years)

SMOKING

13) Do you currently smoke?

☐Yes (___ pack/day x ___years)

☐Previously (___ pack/day x ___years) but stopped for ☐<6m ☐ ≥6m

☐No

14) On average, how many of the following do you smoke a day? (tick all that apply)

☐Cigarettes ____/day

☐Ang hoon ____/day

☐Pipes ____/day

☐Cigars ____/day

15) What is highest number of cigarettes smoked a day?

____

16) Are you currently exposed to cigarette smoke at home?

☐Never ☐Sometimes ☐Most of the time ☐All the time

17) Are you currently exposed to cigarette smoke at work?

☐Never ☐Sometimes ☐Most of the time ☐All the time

ALCOHOL

18) Have you consumed alcohol within the past 3 months?

☐Yes (___ units per week)

☐No

(1 unit= half pint of beer, small glass of wine or shot of spirit/hard liquor)

19) How often in the last year did you drink the following alcoholic beverages? (tick all that apply)

☐Beer can	______ per week
☐Beer bottle	______ per week
☐Red wine	______ glasses per week
☐White wine	______ glasses per week
☐Sparkling wine	______ glasses per week
☐Hard liquor	______ glasses per week

TRADITIONAL/COMPLEMENTARY MEDICINE

20) Do you use Traditional medicines/complementary medicines?

☐Yes

☐No

21) On average, how many times **a month** do you use traditional/complimentary medicines?

_____ times

22) What type of traditional/complementary medicines do you use? (tick all that apply)

☐Traditional Chinese medicine

☐Acupuncture

☐Jamu/Traditional Malay medicine

☐Traditional Indian medicine

☐Relaxation therapies: yoga, meditation

☐Herbal remedies: garlic, ginger, ginkgo biloba etc

☐Vitamins

☐Massages

☐Chiropractic

☐Tai chi or chi gong

☐Magnetic therapy

☐Others: ________

EXERCISE

23) How often do you exercise a week?

☐Never/hardly (ie. not even once a week on average)

☐ ___ times for ____ min each time

Please specify type of exercise: ________________

DIET

24) How much coffee do you drink a week?

☐ never or rarely ☐ <1 cup a week

☐ ≥1 cup a week but <1 daily ☐___ cups a day

(tick all that apply) ☐with milk? ☐with sugar?

25) How much English tea do you drink a week?

☐ never or rarely ☐ <1 cup a week

☐ ≥1 cup a week but <1 daily ☐___ cups a day

(tick all that apply) ☐with milk? ☐with sugar?

26) How much Chinese tea do you drink a week?

☐ never or rarely ☐ <1 cup a week

☐ ≥1 cup a week but <1 daily ☐___ cups a day

(tick all that apply) ☐with milk? ☐with sugar?

27) How much Green tea do you drink a week?

☐ never or rarely ☐ <1 cup a week

☐ ≥1 cup a week but <1 daily ☐___ cups a day

(tick all that apply) ☐with milk? ☐with sugar?

28) How many servings of vegetables do you take a day?

_______ servings

(eg. 1 serving is ¾ mug cooked leafy or non-leafy vegetables (100g)/ ¼ round plate of cooked vegetables/150g raw leafy vegetables/100g raw non-leafy vegetables)

29) How many servings of fruits do you take a day?

_______ servings

(one serving is1 small apple, orange, pear or mango/1 wedge of pineapple, papaya or watermelon/10 grapes or longans (50g)/1 medium banana)

PERCEPTIONS

30) How active do you think your lifestyle is? (Circle your answer)

1	2	3	4	5	6	7	8	9	10
**Not active**	→								**Very active**

31) In general, how stressed do you feel? (Circle your answer)

1	2	3	4	5	6	7	8	9	10
**Not stressed**	→								**Very stressed**

Health Questionnaire

English version for Singapore

Singapore (English) © 2003 EuroQol Group EQ-5D™ is a trade mark of the EuroQol Group

By placing a tick in one box in each group below, please indicate which statements best describe your own health state today.

Mobility

I have no problems in walking about ❑

I have some problems in walking about ❑

I am confined to bed ❑

Self-Care

I have no problems with self-care ❑

I have some problems washing or dressing myself ❑

I am unable to wash or dress myself ❑

Usual Activities *(e.g. work, study, housework, family or leisure activities)*

I have no problems with performing my usual activities ❑

I have some problems with performing my usual activities ❑

I am unable to perform my usual activities ❑

Pain / Discomfort

I have no pain or discomfort ❑

I have moderate pain or discomfort ❑

I have extreme pain or discomfort ❑

Anxiety / Depression

I am not anxious or depressed ❑

I am moderately anxious or depressed ❑

I am extremely anxious or depressed ❑

Singapore (English) © 2003 EuroQol Group EQ-5D™ is a trade mark of the EuroQol Group

To help people say how good or bad a health state is, we have drawn a scale (rather like a thermometer) on which the best state you can imagine is marked 100 and the worst state you can imagine is marked 0.

We would like you to indicate on this scale how good or bad your own health is today, in your opinion. Please do this by drawing a line from the **BLACK BOX** below to whichever point on the scale indicates how good or bad your health state is today.

Your own health state today

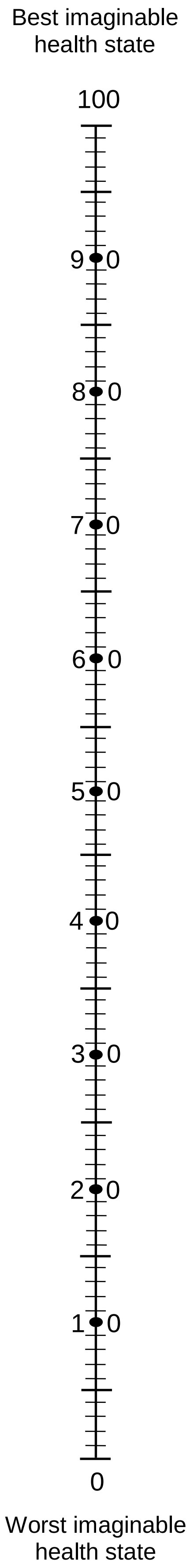

Singapore (English) © 2003 EuroQol Group EQ-5D™ is a trade mark of the EuroQol Group

AM
Subject's Initials________________ID#__________________Date_______________Time_____PM

PITTSBURGH SLEEP QUALITY INDEX

INSTRUCTIONS:
The following questions relate to your usual sleep habits during the past month only. Your answers should indicate the most accurate reply for the majority of days and nights in the past month. Please answer all questions.

1. During the past month, what time have you usually gone to bed at night?

   BED TIME ___________

2. During the past month, how long (in minutes) has it usually taken you to fall asleep each night?

   NUMBER OF MINUTES ___________

3. During the past month, what time have you usually gotten up in the morning?

   GETTING UP TIME ___________

4. During the past month, how many hours of actual sleep did you get at night? (This may be different than the number of hours you spent in bed.)

   HOURS OF SLEEP PER NIGHT ___________

For each of the remaining questions, check the one best response. Please answer all questions.

5. During the past month, how often have you had trouble sleeping because you . . .

a) Cannot get to sleep within 30 minutes

Not during the past month_____ Less than once a week_____ Once or twice a week_____ Three or more times a week_____

b) Wake up in the middle of the night or early morning

Not during the past month_____ Less than once a week_____ Once or twice a week_____ Three or more times a week_____

c) Have to get up to use the bathroom

Not during the past month_____ Less than once a week_____ Once or twice a week_____ Three or more times a week_____

d) Cannot breathe comfortably

Not during the past month_____	Less than once a week_____	Once or twice a week_____	Three or more times a week_____

e) Cough or snore loudly

Not during the past month_____	Less than once a week_____	Once or twice a week_____	Three or more times a week_____

f) Feel too cold

Not during the past month_____	Less than once a week_____	Once or twice a week_____	Three or more times a week_____

g) Feel too hot

Not during the past month_____	Less than once a week_____	Once or twice a week_____	Three or more times a week_____

h) Had bad dreams

Not during the past month_____	Less than once a week_____	Once or twice a week_____	Three or more times a week_____

i) Have pain

Not during the past month_____	Less than once a week_____	Once or twice a week_____	Three or more times a week_____

j) Other reason(s), please describe____

____

How often during the past month have you had trouble sleeping because of this?

Not during the past month_____	Less than once a week_____	Once or twice a week_____	Three or more times a week_____

6. During the past month, how would you rate your sleep quality overall?

Very good ___________

Fairly good ___________

Fairly bad ___________

Very bad ___________

7. During the past month, how often have you taken medicine to help you sleep (prescribed or "over the counter")?

Not during the past month_____	Less than once a week_____	Once or twice a week_____	Three or more times a week_____

8. During the past month, how often have you had trouble staying awake while driving, eating meals, or engaging in social activity?

Not during the past month_____	Less than once a week_____	Once or twice a week_____	Three or more times a week_____

9. During the past month, how much of a problem has it been for you to keep up enough enthusiasm to get things done?

No problem at all	__________
Only a very slight problem	__________
Somewhat of a problem	__________
A very big problem	__________

10. Do you have a bed partner or room mate?

No bed partner or room mate	__________
Partner/room mate in other room	__________
Partner in same room, but not same bed	__________
Partner in same bed	__________

If you have a room mate or bed partner, ask him/her how often in the past month you have had . . .

a) Loud snoring

Not during the past month_____	Less than once a week_____	Once or twice a week_____	Three or more times a week_____

b) Long pauses between breaths while asleep

Not during the past month_____	Less than once a week_____	Once or twice a week_____	Three or more times a week_____

c) Legs twitching or jerking while you sleep

Not during the past month_____	Less than once a week_____	Once or twice a week_____	Three or more times a week_____

d) Episodes of disorientation or confusion during sleep

Not during the past month_____	Less than once a week_____	Once or twice a week_____	Three or more times a week_____

e) Other restlessness while you sleep; please describe________________________________

____

Not during the past month_____	Less than once a week_____	Once or twice a week_____	Three or more times a week_____

© 1989, University of Pittsburgh. All rights reserved. Developed by Buysse,D.J., Reynolds,C.F., Monk,T.H., Berman,S.R., and Kupfer,D.J. of the University of Pittsburgh using National Institute of Mental Health Funding.

Buysse DJ, Reynolds CF, Monk TH, Berman SR, Kupfer DJ: Psychiatry Research, 28:193-213, 1989.

For Men Only

<u>International Index of Erectile Function (IIEF)</u>

<u>Please use an X where applicable and be sure to initial and date all corrections</u>

Score 0 if not done   Subject questionnaire Section 1

Instructions: These questions ask about the effects your erection problems have had on your sex life, over the past 4 weeks. Please answer the following questions as honestly and clearly as possible. In answering these questions, the following definitions apply:

Definitions:

- *Sexual activity includes intercourse, caressing, foreplay and masturbation*
- *Sexual intercourse is defined as vaginal penetration of the partner (you entered the partner)*
- *Sexual stimulation includes situations like foreplay with a partner, looking at erotic pictures, etc.*
- *Ejaculate is defined as the ejection of semen from* the penis (or the feeling of this)

Mark ONLY one circle per question:

1. Over the past 4 weeks, how often were you able to get an erection during sexual activity?

□ No sexual activity
□ Almost always or always
□ Most times (much more than half the time)
□ Sometimes (about half the time)
□ A few times (much less than half the time)
□ Almost never or never

2. Over the past 4 weeks, when you had erections with sexual stimulation, how often were your erections hard enough for penetration?

□ No sexual stimulation
□ Almost always or always
□ Most times (much more than half the time)
□ Sometimes (about half the time)
□ A few times (much less than half the time)
□ Almost never or never

Questions 3, 4 and 5 will ask about erections you may have had during sexual intercourse.

3. Over the past 4 weeks, when you attempted sexual intercourse, how often were you able to penetrate (enter) your partner?

□ Did not attempt intercourse
□ Almost always or always
□ Most times (much more than half the time)
□ Sometimes (about half the time)
□ A few times (much less than half the time)
□ Almost never or never

4. Over the past 4 weeks, during sexual intercourse, how often were you able to maintain your erection after you had penetrated (entered) your partner?

□ Did not attempt intercourse
□ Almost always or always
□ Most times (much more than half the time)
□ Sometimes (about half the time)
□ A few times (much less than half the time)
□ Almost never or never

5. Over the past 4 weeks, during sexual intercourse, how difficult was it to maintain your erection to completion of intercourse?

□ Did not attempt intercourse
□ Extremely difficult
□ Very difficult
□ Difficult
□ Slightly difficult
□ Not difficult

6. Over the past 4 weeks, how many times have you attempted sexual intercourse?

□ No attempts
□ 1-2 attempts
□ 3-4 attempts
□ 5-6 attempts
□ 7-10 attempts
□ 11 or more attempts

7. Over the past 4 weeks, when you attempted sexual intercourse how often was it satisfactory for you?

□ Did not attempt intercourse
□ Almost always or always
□ Most times (much more than half the time)
□ Sometimes (about half the time)
□ A few times (much less than half the time)
□ Almost never or never

8. Over the past 4 weeks, how much have you enjoyed sexual intercourse?

□ No intercourse
□ Very highly enjoyable
□ Highly enjoyable
□ Fairly enjoyable
□ Not very enjoyable
□ Not enjoyable

9. Over the past 4 weeks, when you had sexual stimulation or intercourse how often did you ejaculate?

□ Did not attempt intercourse
□ Almost always or always
□ Most times (more than half the time)
□ Sometimes (about half the time)
□ A few times (much less than half the time)
□ Almost never or never

10. Over the past 4 weeks, when you had sexual stimulation or intercourse how often did you have the feeling of orgasm or climax (with or without ejaculation)?

□ No sexual stimulation or intercourse
□ Almost always or always
□ Most times (much more than half the time)
□ Sometimes (about half the time)
□ A few times (much less than half the time)
□ Almost never or never

Questions 11 and 12 ask about sexual desire. Let's define sexual desire as a feeling that may include wanting to have a sexual experience (for example, masturbation or intercourse), thinking about having sex or feeling frustrated due to a lack of sex.

11. Over the past 4 weeks, how often have you felt sexual desire?

□ Almost always or always
□ Most times (much more than half the time)
□ Sometimes (about half the time)
□ A few times (much less than half the time)
□ Almost never or never

12. Over the past 4 weeks, how would you rate your level of sexual desire?

□ Very high
□ High
□ Moderate
□ Low
□ Very low or none at all

13. Over the past 4 weeks, how satisfied have you been with you overall sex life?

□ Very satisfied
□ Moderately satisfied
□ About equally satisfied and dissatisfied
□ Moderately dissatisfied
□ Very dissatisfied

14. Over the past 4 weeks, how satisfied have you been with your sexual relationship with your partner?

□ Very satisfied
□ Moderately satisfied
□ About equally satisfied and dissatisfied
□ Moderately dissatisfied
□ Very dissatisfied

15. Over the past 4 weeks, how do you rate your confidence that you can get and keep your **erection**?

□ Very high
□ High
□ Moderate
□ Low
□ Very low
